# Yifei Sanjie Formula Treats Chronic Obstructive Pulmonary Disease by Remodeling Pulmonary Microbiota

**DOI:** 10.3389/fmed.2022.927607

**Published:** 2022-06-29

**Authors:** Yueying Wu, Hui Meng, Bo Qiao, Ning Li, Qiang Zhang, Wenqing Jia, Haijing Xing, Yuqing Li, Jiali Yuan, Zhongshan Yang

**Affiliations:** ^1^Yunnan Provincial Key Laboratory of Molecular Biology for Sinomedicine, Kunming, China; ^2^First Clinical School of Medicine, Yunnan University of Chinese Medicine, Kunming, China; ^3^Basic Medical School, Shanghai University of Chinese Medicine, Shanghai, China

**Keywords:** chronic obstructive pulmonary disease, pulmonary microbiota, inflammation, Yifei Sanjie Formula, NLRP3/caspase-1/IL-1β signaling pathway

## Abstract

Chronic obstructive pulmonary disease (COPD) is one of the most common pulmonary diseases. Evidence suggests that dysbiosis of pulmonary microbiota leads to the COPD pathological process. Yifei Sanjie Formula (YS) is widely used to treat diseases in respiratory systems, yet little is known about its mechanisms. In the present study, we first established the fingerprint of YS as the background for UHPLC-QTOF-MS. Components were detected, including alkaloids, amino acid derivatives, phenylpropanoids, flavonoids, terpenoids, organic acids, phenols, and the like. The therapeutic effect of YS on COPD was evaluated, and the pulmonary function and ventilatory dysfunction (EF50, TV, and MV) were improved after the administration of YS. Further, the influx of lymphocytes was inhibited in pulmonary parenchyma, accompanied by down-regulation of inflammation cytokines *via* the NLRP3/caspase-1/IL-1β signaling pathway. The severity of pulmonary pathological damage was reversed. Disturbed pulmonary microbiota was discovered to involve an increased relative abundance of *Ralstonia* and *Mycoplasma* and a decreased relative abundance of *Lactobacillus* and *Bacteroides* in COPD animals. However, the subversive effect was shown. The abundance and diversity of pulmonary microflora were remodeled, especially increasing beneficial genua *Lactobacillus* and *Bacteroides*, as well as downregulating pathogenic genua *Ralstonia* and *Mycoplasma* in the YS group. Environmental factor correlation analysis showed that growing pulmonary microbiota was positively correlated with the inflammatory factor, referring to *Ralstonia* and *Mycoplasma*, as well as negatively correlated with the inflammatory factor, referring to *Lactobacillus* and *Bacteroides*. These results suggest that the effects of YS involved remodeling lung microbes and anti-inflammatory signal pathways, revealing that intervention microbiota and an anti-inflammatory may be a potential therapeutic strategy for COPD.

## Introduction

Chronic obstructive pulmonary disease (COPD) is a complex respiratory system disease characterizing continuous respiratory symptoms and airflow limitation followed by a progressive and irreversible decline in lung function. This disease has become the third-most-common life-threatening disease worldwide, leading to substantial social and economic burdens (1, 2). The complex pathogenesis of COPD includes inflammation, oxidative stress, and protease/antiprotease imbalance (3, 4). The inflammatory immune response runs throughout the whole disease process of COPD to reduce inflammation. It is beneficial in delaying the development and improving symptoms of COPD (5). Chronic inflammation in COPD is attributed to the infiltration of various immune cells, macrophages, lymphocytes, neutrophils, and the like, leading to epithelial cell injury and fibrocyte activation to remodel the airway structure (6). The nod-like receptor protein 3 (NLRP3) is known as a potent inducer of inflammation to process Interleukin-1β (IL-1β) and IL-18 (7, 8). NLRP3 inflammasome is essential for the development of COPD in animal or clinical research of human respiratory system samples (9, 10). Activating NLRP3 may be initiate the inflammatory response by binding to cytoplasmic pathogen-associated molecular patterns or damage-associated molecular patterns to aggravate COPD (11). Targeted anti-inflammatory therapy is likely to benefit COPD (12).

In the lungs and airways, there are enormous and complicated microbes that have a profound effect on the host's health, nutrition, and disease progression (13). Microbial community structure and distribution are crucial in maintaining a homeostasis environment (14). Research suggests that bacteria in the respiratory tract resist colonization by foreign pathogens, and cigarette smoking alters the composition of the pulmonary microbiota (15). Pulmonary microbiota dysbiosis is related to inflammation, pathological airway alterations, immune responses, and the aggravation of clinical symptoms in patients with COPD (16). Pathogens stimulate inflammatory cells to produce inflammatory media that often destroy the immune function of the airway and mucosa, leading to chronic inflammation and pulmonary microbiota dysbiosis, further aggravating COPD (17). Undeniably, remodeling pulmonary microbiota structure and proportion is a novel strategy for treating COPD.

**Figure 1:**
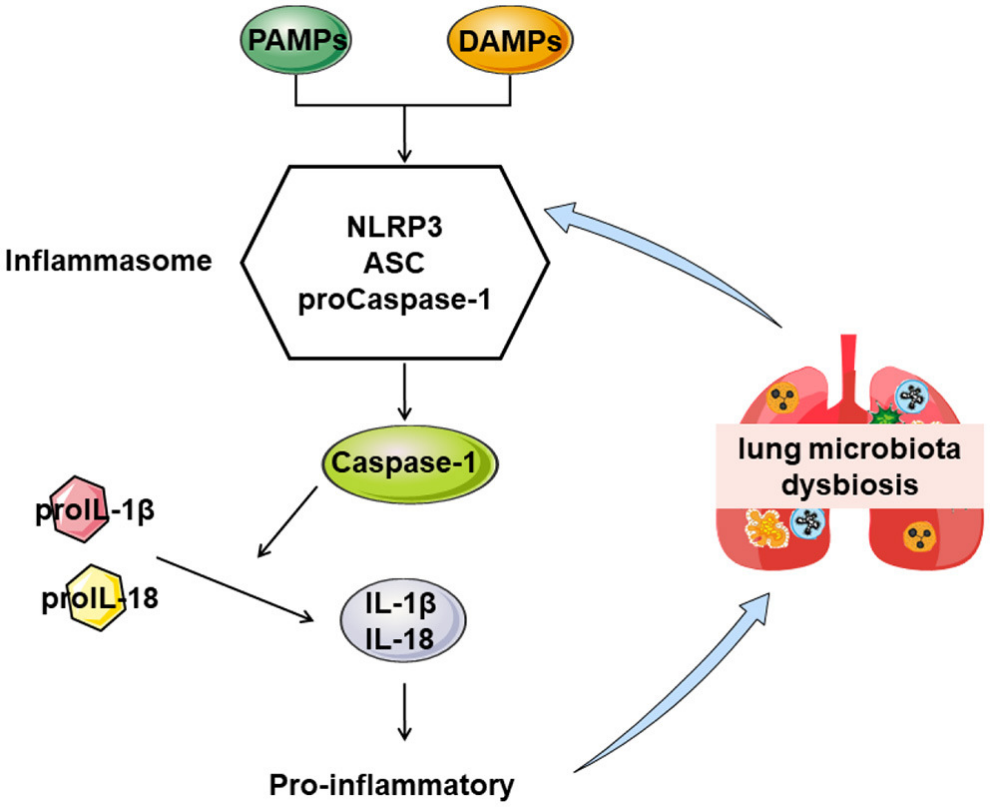
The proposed mechanism by which YS treats COPD.

Much attention has been paid to the development of Chinese herbal medicines to treat COPD (18). Yifei Sanjie Formula (YS), a traditional Chinese medicine, comprises eight medicinal herbs [*Hedysarum Multijugum Maxim, Atractylodes Macrocephala Koidz, Saposhnikoviae Radix, Sinapis Semen, Fritillariae Thunbrgii Bulbus, Mori Cortex, Curcumae Rhizoma*, and *Panax Notoginseng (Burk.) F. H. Chen Ex C. Chow*] and has been shown to possess extensive pharmacological effects against pulmonary disease, including the reduction of lung injury and inflammatory responses (19, 20). Nevertheless, the pharmacological mechanisms of YS on COPD remain poorly understood. Therefore, we explored the efficacy mechanism of YS on COPD in alleviating lung injury and the ventilatory function and the relationship between the efficacy of YS and pulmonary microflora. We aimed to support YS's efficacy in correcting pulmonary microbiota dysbiosis (Figure 1).

## Materials and Methods

### Groups and Treatments

**Figure 2:**
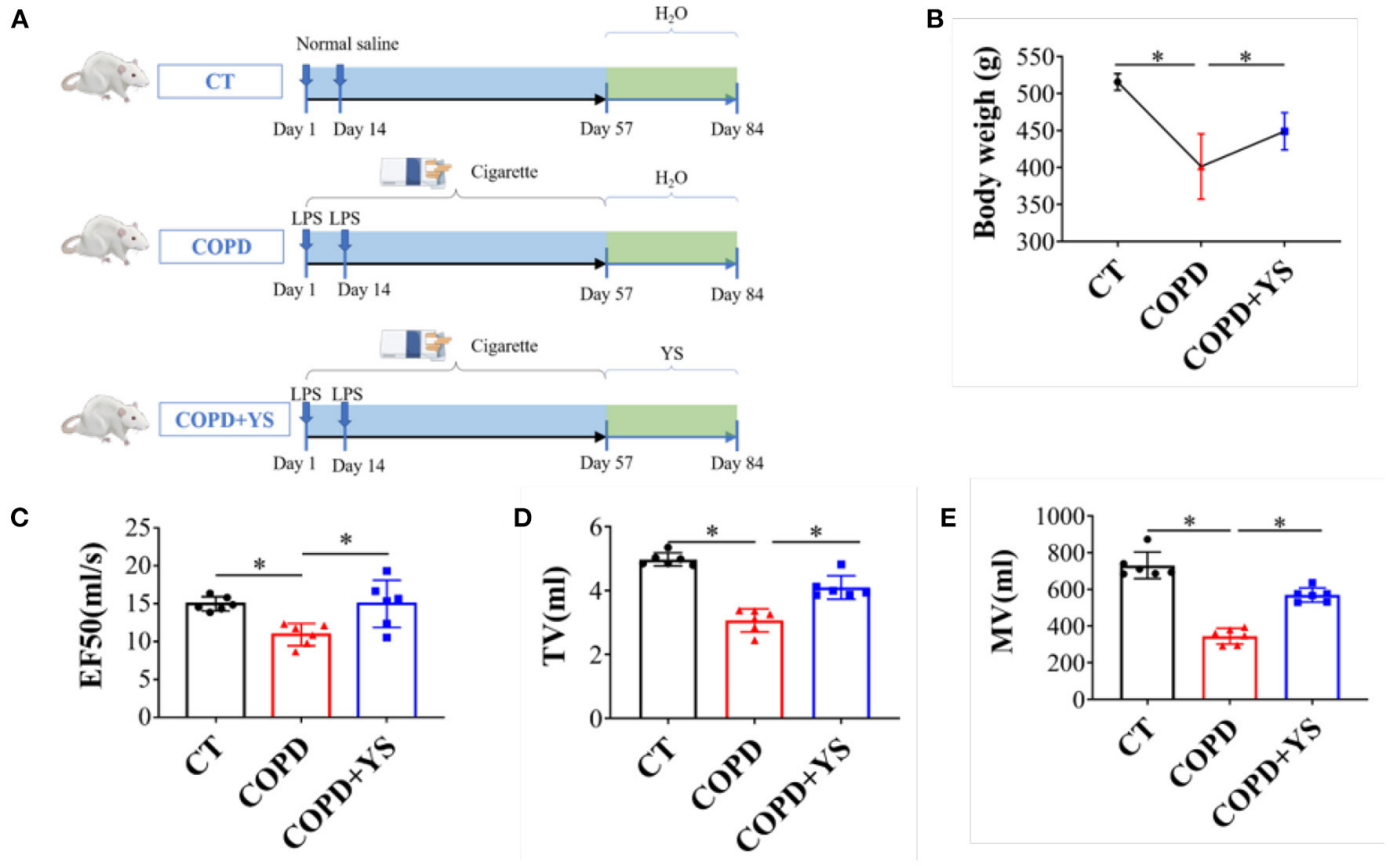
**(A)** Timeline diagrams of the experimental design (*n* = 6). **(B)** Body weight of the three groups including CT, COPD, and COPD + YS (*n* = 6). **(C)** EF50 of CT, COPD, and COPD + YS (*n* = 6). **(D)** TV of CT, COPD and COPD + YS (*n* = 6). **(E)** MV of CT, COPD and COPD+ YS (*n* = 6). The data are presented as the means ± SEM of three experiments (*P < 0.05).

According to the “Guidelines for the Care and Use of Laboratory Animals” published by the National Institutes of Health, all rat experiments were approved by the Animal Ethics Experiment Committee of Yunnan University of Traditional Chinese Medicine and were conducted following the guidelines of the committee. SPF Wistar rats (*n* = 18; 200 ± 20 g; male) were provided by Chengdu Dossy Experimental Animals Co., Ltd. [Chengdu, China; license No. SCXK (Chuan)−2020-030]. The rats were randomly divided into the control (CT), COPD, and COPD + YS groups. Besides the CT group, the rat COPD model was replicated by using cigarette smoke exposure (Hongyun cigarette, tar 11 mg, nicotine 1.1 mg, CO12 mg) combined with airway instillation of lipopolysaccharide (LPS, Sigma) (21). 11.6 g/kg once per day of YS was administered daily by intragastric administration from days 57 to 84 (the same dosage is used clinically) (Figure 2A). Animal weight and behaviorism were monitored regularly and recorded. The composition of YS is shown in Table 1.

**Table 1:** Prescription of YS.

**NO**.	**Chinese Pinyin name**	**Latin name**
1	Huangqi	*Hedysarum Multijugum Maxim*
2	Baizhu	*Atractylodes Macrocephala Koidz*
3	Fangfeng	*Saposhnikoviae Radix*
4	Jiezi	*Sinapis Semen*
5	Zhe Beimu	*Fritillariae Thunbrgii Bulbus*
6	Sang Baipi	*Mori Cortex*
7	Ezhu	*Curcumae Rhizoma*
8	Sanqi	*Panax Notoginseng (Burk.) F. H. Chen Ex C. Chow*

### Pulmonary Function Testing

Pulmonary function testing was performed with a whole-body plethysmograph (EMMS, Alton, Hants, UK) after the experiment was over (12 weeks). According to the whole-body plethysmograph, data for EF50 (Expiratory Flow at 50%), tidal volume (TV), and minute volume (MV) were collected. All of the raw data were obtained from Supplementary Material S17.

### Histological Evaluation

The upper lobe of the right lung of the rat was fixed with 4% formalin for 48 h and then embedded in paraffin, stained with hematoxylin and eosin (H&E) and Masson. Finally, it was evaluated histopathologically under an optical microscope (OLYMPUS VS200). All of the full scans of the entire original gels are displayed in Supplementary Materials S1–12.

### Serum Cytokine/Protein Levels

Protein was extracted from lung tissue. Protein quantification was performed using the BCA protein quantification kit (P0012, Beyotime, Jiangsu, China) to calculate loading capacity. Samples were added to a precast SDS polyacrylamide gel for electrophoretic separation, and proteins in the gel were electrotransferred to a PVDF membrane using water bath electroblotting. The primary antibodies were added, anti-TGF-β1 (rabbit polyclonal antibody, Abcam, ab179695) and anti-NLRP3 (rabbit polyclonal antibody, Bioss, bs-10021R), overnight at 4°C. After washing the membrane, a secondary antibody was added, shook at 4°C, and incubated for 1 h. PVDF was dropped, and ECL luminescence liquid was developed and photographed (GeneGnome). The grayscale values of individual bands were analyzed using gene tools software. All of the full scans of the entire original gels are displayed in S13 to S16. ELISA kit (Jiangsu Enzyme-Linked Biotechnology Co., Ltd.) was used to detect the concentration of TNF-α and IL-6 in serum and the concentration of IL-1β, IL-18, and SIgA in the lung. The method and procedure were completed according to the kit operating instructions. Finally, read the absorbance at 450 nm after adding the stop solution within 15 min. All of the raw data were obtained from Supplementary Material S17.

### 16S rRNA Gene High-Throughput Sequencing to Detect the Pulmonary Microbiota

The total genome DNA from samples of the pulmonary microbiota was extracted using the CTAB/SDS method. DNA concentration and purity were monitored on 1% agarose gels. According to the concentration, DNA was diluted to 1 ng/μL using sterile water. 16S rRNA genes of distinct regions (16S V4/16S V3/16S V3-V4) were amplified using a specific primer (16S V4:515F-806R) with the barcode. PCR products were mixed in equidensity ratios. Then, the mixture of PCR products was purified with Qiagen Gel Extraction Kit (Qiagen, Germany). Sequencing libraries were generated using TruSeq® DNA PCR-Free Sample Preparation Kit (Illumina, USA) following the manufacturer's recommendations, and index codes were added. The library quality was assessed on the Qubit@ 2.0 Fluorometer (Thermo Scientific) and AgilentBioanalyzer 2100 system. Finally, the library was sequenced on an Illumina NovaSeq platform, and 250 bp paired-end reads were generated. The data on pulmonary microbiota were obtained from the online tool-NovoMagic at https://magic.novogene.com *via* a personal account. All of the raw data were obtained from Supplementary Material S17.

### UHPLC-QE-MS to Screen the Components of YS

We used UHPLC-QE-MS to detect and analyze the potential active components of YS to exert its medicinal effect and further clarify the components of YS. LC-MS/MS analysis was performed on a UHPLC system (Vanquish, Thermo Fisher Scientific) with a Waters UPLC BEH C18 column (1.7 μm 2.1 ^*^100 mm). The flow rate was set at 0.4 mL/min, and the sample injection volume was set at 5 μL. The mobile phase consisted of 0.1% formic acid in water (A) and 0.1% formic acid in acetonitrile (B). The multi-step linear elution gradient program was as follows: 0–3.5 min, 95–85% A; 3.5–6 min, 85–70% A; 6–6.5 min, 70–70% A; 6.5–12 min, 70–30% A; 12–12.5 min, 30–30% A; 12.5–18 min, 30–0% A; 18–25 min, 0–0% A; 25–26 min, 0–95% A; 26–30 min, 95–95% A.

An Orbitrap Exploris 120 mass spectrometer coupled with an Xcalibur software was employed to obtain the MS and MS/MS data based on the IDA acquisition mode. During each acquisition cycle, the mass range was from 100 to 1,500, and the top four of every cycle were screened. The corresponding MS/MS data were further acquired. Sheath gas flow rate: 30 Arb, Aux gas flow rate: 10 Arb, Ion Transfer Tube Temp: 350°C, Vaporizer Temp: 350°C, Full ms resolution: 60,000, MS/MS resolution: 15,000, Collision energy: 16/38/42 in NCE mode, Spray Voltage: 5.5 kV (positive) or−4 kV (negative).

### Statistical Analysis

Data were expressed as means ± standard deviation ($\bar{x}$ ±s). The normal distribution of the data was assessed with the Shapiro-Wilk test. Depending on the data normality distribution, significant differences in the variance of parameters were evaluated either with ANOVA or the Kruskal-Wallis test in SPSS 22.0. All statistical tests were two-sided, and a *P-*value of < 0.05, or FDR adjusted *P*adj value < 0.05 was considered statistically significant. The analysis of pulmonary microbiota was performed using the online tool-NovoMagic at https://magic.novogene.com *via* a personal account. All raw data were obtained from S17 and the file of statistical programs (original).

## Results

### YS Improved Pulmonary Function in COPD Rats

The pulmonary function is an important index in evaluating COPD, including TV, MV, and EF50, reflecting a small airway obstruction. Studies found that COPD patients had decreased TV and MV. Our data showed that EF50, TV, and MV were significantly lower in COPD rats (*P* < 0.05), while EF50, TV, and MV were higher after YS intervention (*P* < 0.05) (Figures 2C–E). COPD rats exhibited hair withering, slow action, and shortness of breath. There were significant improvements in animal behaviors after treatment with YS. The body weights of COPD rats significantly decreased (*P* < 0.05). And, the body weights in the YS+ COPD group significantly increased (*P* < 0.05) (Figure 2B). These findings suggest that YS improves animal behaviors and pulmonary ventilatory functions in COPD rats.

### Screening Components of YS With UHPLC-QTOF-MS

**Figure 3:**
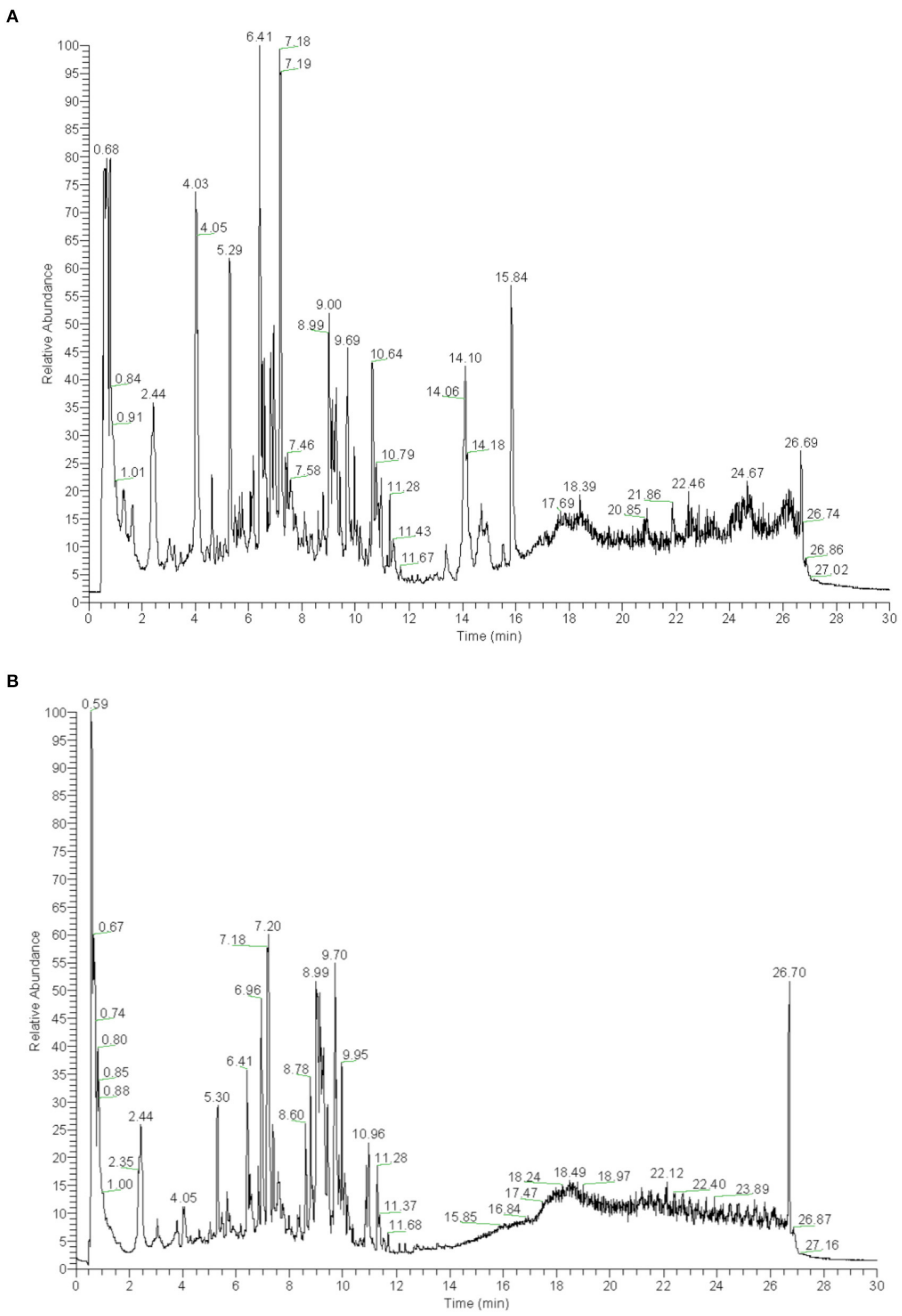
**(A)** UHPLC-QTOF-MS analysis base peak intensity chromatograms of YS in positive mode. **(B)** UHPLC-QTOF-MS analysis base peak intensity chromatograms of YS in negative mode.

YS is a Chinese herbal medicine formula. It is composed of *Hedysarum Multijugum Maxim, Atractylodes macrocephala Koidz, Saposhnikoviae Radix, Sinapis Semen, Fritillaria Thunbrgii Bulbus, Mori Cortex, Curcumae Rhizoma* and *Panax Notoginseng (Burk.) F. H. Chen Ex C. Chow*. We performed UHPLC-QTOF-MS to screen for the components of YS. We established the fingerprint of YS as the background for UHPLC-QTOF-MS, including the positive ion mode and negative ion mode. We analyzed the top 20 components by peak areas with the main peaks separated (Figures 3A,B). Among these, eight kinds of components were detected, including alkaloids, amino acid derivatives, carbohydrates and their derivatives, carboxylic acids and their derivatives, phenylpropanoids, flavonoids, terpenoids, organic acids and their derivatives, phenols, and aliphatic acyl based on a comparison with standard materials (Table 2). The main active products may be one or more of these components.

**Table 2:** Screening the top 20 components of YS with UHPLC-QTOF-MS.

**No**.	**Components**	**CompositeScore**	**Class**	**Electric**	**Rtmed(s)**	**Peak area**
1	4-Hydroxybenzoylcholine	0.831	Alkaloid	+H	98.380	4E+09
2	Arginine	0.984	Amino acid derivatives	+H	36.876	2.8E+09
3	4-Amino-2-methylenebutanoic acid	0.998	Others	+H	41.834	2.7E+09
4	SUCROSE	0.688	Carbohydrates and derivatives	–H	38.728	2E+09
5	Citric acid	0.990	Carboxylic acids and derivatives	–H	44.239	1.8E+09
6	Adenosine	1.000	Alkaloid	+H	48.666	1.6E+09
7	p-Coumaric acid	1.000	Phenylpropanoids	+H	98.380	1.5E+09
8	Phenylalanine	0.976	Amino acid derivatives	+H	80.181	1.5E+09
9	Prunetin	0.987	Flavonoids	+H	454.896	1.3E+09
10	Biochanin-7-O-glucoside	0.911	Flavonoids	+H	339.706	1.2E+09
11	Isoleucine	0.967	Amino acid derivatives	+H	55.474	1.2E+09
12	Ginsenoside F1	0.860	Terpenoids	–H	556.663	1.E+09
13	N2-Fructopyranosylarginine	1.000	Others	+H	36.630	8.1E+08
14	2-Chloro-DL-Phenylalanine	1.000	Internal standards	+H	150.892	8E+08
15	Tectochrysin	0.972	Flavonoids	+H	559.658	6.1E+08
16	Acacetin	0.990	Flavonoids	–H	390.813	5.5E+08
17	O-Methylcorypalline	0.969	Alkaloid	+H	207.566	5.4E+08
18	Ginsenoside Re	0.958	Terpenoids	–H	429.754	5.3E+08
19	Formononetin-7-O-glucoside	0.610	Flavonoids	+H	429.358	4.9E+08
20	D-Gluconic acid	0.958	Organic acids and derivatives	–H	38.028	4.5E+08
21	Curdione	0.986	Terpenoids	+H	671.712	4.1E+08
22	3-Hydroxy-5-isopropylidene-3,8-dimethyl-2,3,3a,4,5,8a-hexahydro-6(1H)-azulenone	0.987	Terpenoids	+H	403.394	4.1E+08
23	8-(2-hydroxy-1-methoxy-3-methylbut-3-enyl)-7-methoxychromen-2-one	0.922	Phenylpropanoids	+H	446.978	4E+08
24	Formononetine	0.999	Flavonoids	–H	560.193	3.5E+08
25	Ginsenoside Rf	0.991	Terpenoids	–H	528.602	3.2E+08
26	ADENOSINE 3′,5′-CYCLIC MONOPHOSPHATE	0.900	Alkaloid	+H	44.421	3.1E+08
27	Adenine	1.000	Alkaloid	+H	48.666	3E+08
28	p-Hydroxybenzyl glucosinolate	0.638	Others	–H	43.373	2.9E+08
29	3,4,5-trimethoxycinnamic acid	0.952	Phenylpropanoids	+H	146.417	2.7E+08
30	2-AMINO-2-METHYLPROPANOATE	0.633	Organic acids and derivatives	–H	146.038	2E+08
31	5-OXO-D-PROLINE	0.978	Amino acid derivatives	–H	48.733	1.8E+08
32	Salicylic acid	0.978	Phenols	–H	384.737	1.8E+08
33	MANNITOL	0.991	Others	–H	38.283	1.7E+08
34	Formononetin-7-O-glucoside	0.645	Flavonoids	–H	428.785	1.3E+08
35	Glutaric acid	0.967	Organic acids and derivatives	–H	89.709	1.2E+08
36	L-ASPARAGINE	0.963	Amino acid derivatives	–H	34.140	1.2E+08
37	Inositol	0.789	Others	–H	38.473	1.1E+08
38	2-METHYLMALEATE	0.968	Aliphatic acyl	–H	61.769	9.1E+07
39	Sativanone	0.684	Others	–H	485.593	8.5E+07
40	Azuleno(5,6-c)furan-1(3H)-one, 4,4a,5,6,7,7a,8,9-octahydro-3,4,8-trihydroxy-6,6,8-trimethyl	0.720	Terpenoids	–H	217.031	7.8E+07

### YS Improved Immune Function in COPD Rats

**Figure 4:**
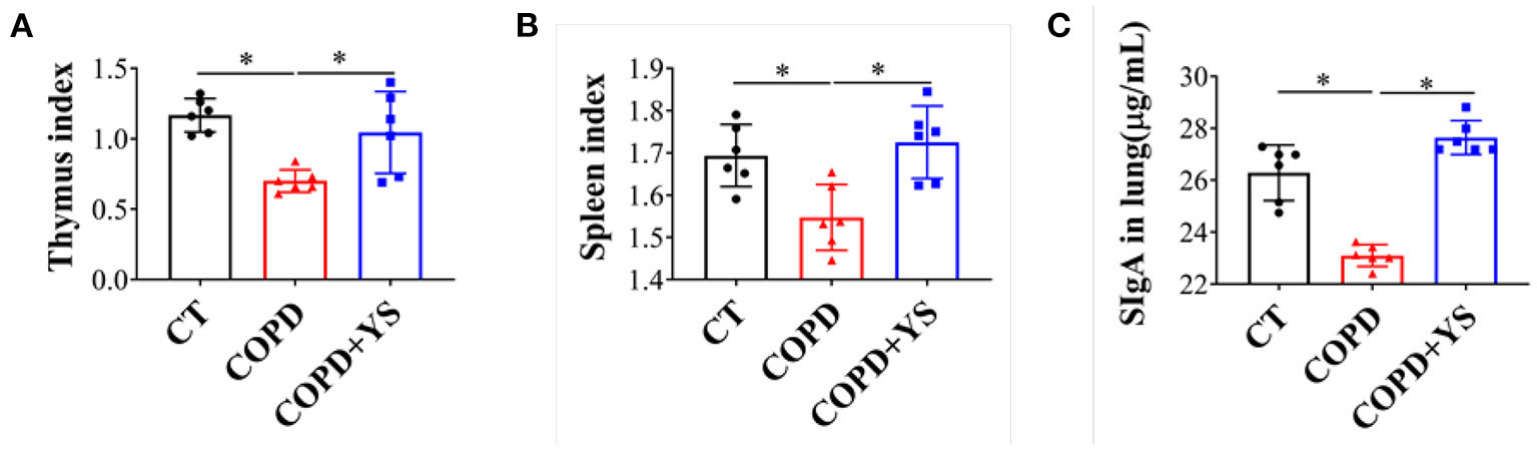
**(A)** Thymus index of CT, COPD, and COPD + YS (*n* = 6). **(B)** Spleen index of CT, COPD, and COPD + YS(*n* = 6). **(C)** The SIgA in the lung of CT, COPD, and COPD + YS (*n* = 6). The data are presented as the means ± SEM of three experiments (*P < 0.05).

The spleen and thymus are critical immune organs, and their organ indexes reflect the strength of immune function to a certain extent (22). Our results showed that the thymus index and spleen index were decreased significantly in COPD rats (*P* < 0.05); after being treated with YS, the thymus and spleen indexes significantly increased (*P* < 0.05) (Figures 4A,B). SIgA is a major effector molecule of the mucosal immune defense system against the colonization and adhesion of pathogenic microorganisms on mucosal surfaces (23). Local SIgA deficiency on small airway surfaces is associated with COPD progression (24). In this present study, the SIgA was decreased in COPD rats (*P* < 0.05), whereas the SIgA in the COPD + YS group was significantly increased (*P* < 0.05) (Figure 4C). These data suggest that YS could enhance the local mucosal immunity and improve immune function in COPD rats.

### YS Ameliorated Inflammation in COPD Rats

**Figure 5:**
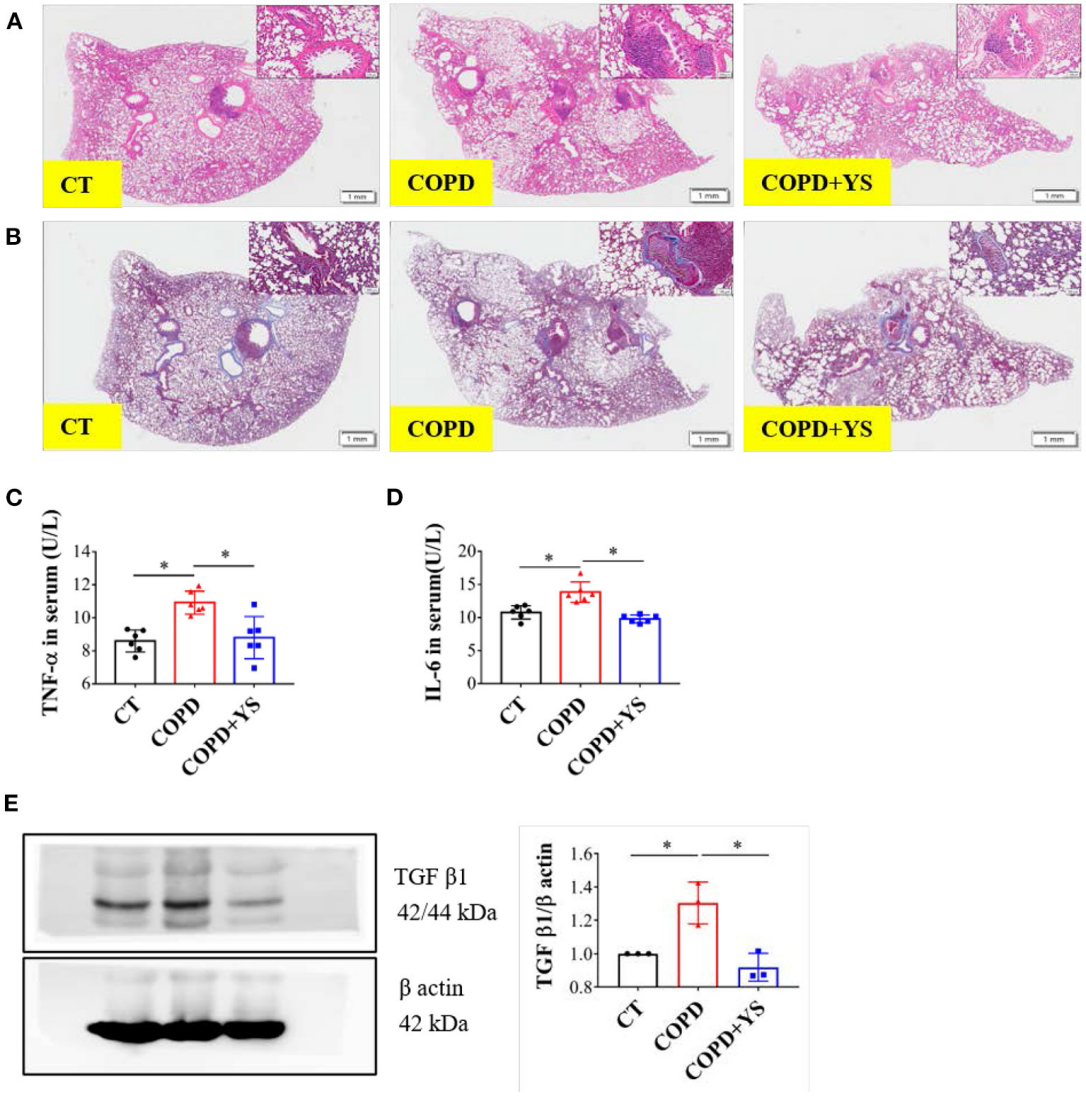
**(A)** H&E staining of lung tissues of CT, COPD, and COPD + YS. **(B)** Masson staining of lung tissues of CT, COPD, and COPD + YS. **(C)** TNF-α in serum of CT, COPD, and COPD + YS (*n* = 6). **(D)** IL-6 in serum of CT, COPD, and COPD + YS (*n* = 6). **(E)** The relative expression of TGF-β1 in the lung of CT, COPD, and COPD + YS (*n* = 3). The data are presented as the means ± SEM of three experiments (*P < 0.05).

The inflammatory response of COPD primarily involves neutrophil infiltration. TNF-α activates neutrophils, and IL-6 inhibits apoptosis (25). HE staining showed that substantial amounts of neutrophil infiltration plugged the bronchi. The lumens were significantly narrowed, and the alveolar walls became thinner and fused in COPD rats. There were fewer pathological changes in the YS intervention group (Figure 5A). Masson staining exhibited that much collagen fiber deposition occurred around the trachea and pulmonary interstitium in COPD rats. Collagen deposition was less severe in COPD+YS rats than in COPD rats (Figure 5B). The levels of TNF-α and IL-6 were significantly higher in the serum of COPD rats (*P* < 0.05) with YS intervention (Figures 5C,D). TGF-β1 is a powerful profibrotic cytokine that participates in inflammatory repair (26). The expression of TGF-β1 was significantly elevated in lung tissue of COPD rats (*P* < 0.05). TGF-β1 in the COPD + YS groupwas significantly lower (*P* < 0.05) (Figure 5E). These findings suggest that YS ameliorates lung tissue damage *via* attenuating inflammatory responses and reducing collagen deposition in COPD rats.

### YS Reduced NLRP3/Caspase-1/IL-1β Signaling Expression in COPD Rats

**Figure 6:**
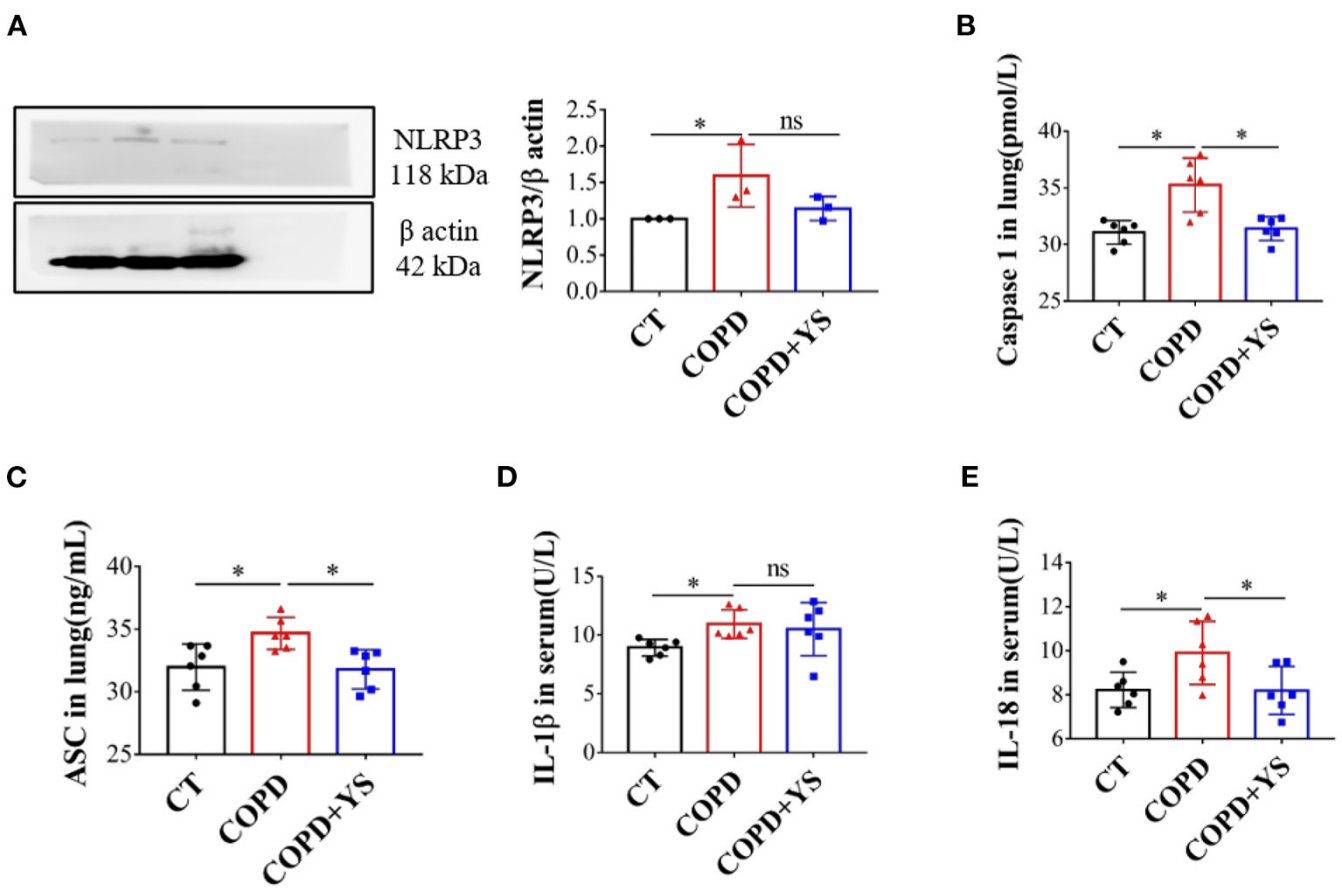
**(A)** NLRP3 in the lung of CT, COPD, and COPD + YS (*n* = 3). **(B)** Caspase1 in the lung of CT, COPD, and COPD + YS (*n* = 6). **(C)** ASC in the lung of CT, COPD, and COPD + YS (*n* = 6). **(D)** IL-1β in the serum of CT, COPD, and COPD+YS (*n* = 6). **(E)** IL-18 in the serum of CT, COPD, and COPD + YS (*n*=6). The data are presented as the means ± SEM of three experiments (ns, non-significant, *P < 0.05).

NLRP3 promotes the release of downstream inflammatory cytokines, inducing acute and chronic inflammatory responses. When NLRP3 is overactivated, ASC acts as an adaptor protein to recruit the precursor caspase-1, promotes IL-1β and IL-18 secreted outside the cell, and exerts proinflammatory effects (27). Compared with control, the protein levels of NLRP3 were significantly greater in lung tissues of COPD rats (*P* < 0.05). Caspase-1 and ASC were significantly increased in lung tissues (*P* < 0.05), and IL-1β and IL-18 were significantly higher in serum (*P* < 0.05). In the COPD + YS group, NLRP3 protein expression was lower in the lung (*P* > 0.05), caspase-1 and ASC were lower in lung tissue (*P* > 0.05), and IL-18 levels of serum were significantly lower (*P* < 0.05). These results suggest that YS inhibits the NLRP3/caspase-1/IL-1β signaling pathway (Figures 6A–E) to alleviate the inflammatory response.

### Effect of YS on Pulmonary Microbiota in COPD Rats

**Figure 7:**
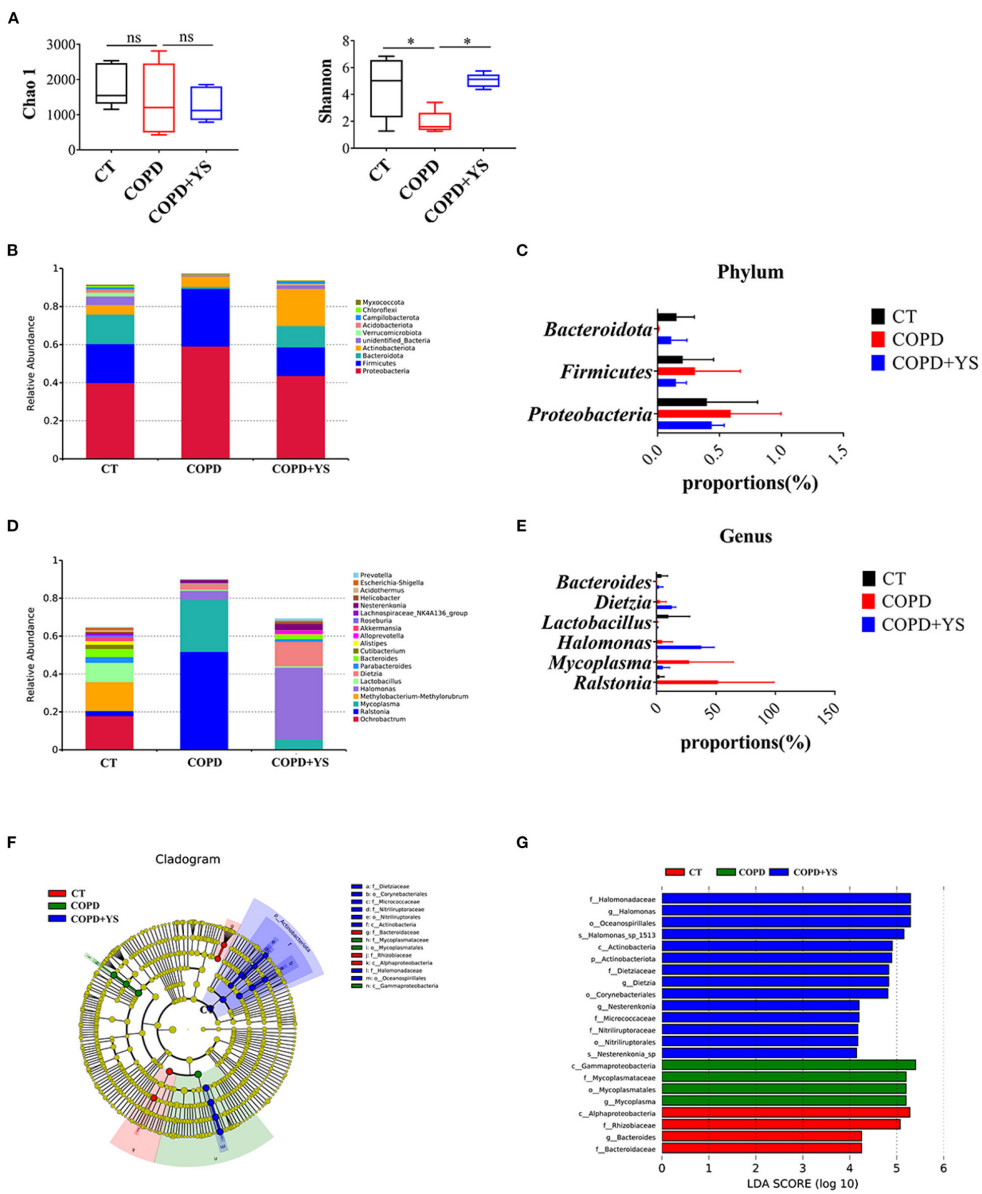
**(A)** Analysis of alpha diversity: Chao1 and Shannon index (*n* = 5). **(B)** The relative abundance of top ten pulmonary microbiota in the phylum (*n* = 5). **(C)** The advantage of pulmonary microbiota in the phylum (*n* = 5). **(D)** The relative abundance of the top twenty pulmonary microbiota in the genera (*n* = 5). **(E)** The advantage of pulmonary microbiota in the genera (*n* = 5). **(F)** Taxonomic differences of pulmonary microbiota among different groups. The taxonomic cladogram was obtained by LEfSe. Differences are represented by the color of the most abundant class. The diameter of each circle is proportional to the taxon's abundance (*n* = 5). **(G)** Linear discriminant analysis score of each group of gut microbiota (*n* = 5). The data are presented as the means ± SEM of three experiments (ns, non-significant, *P < 0.05). The analysis of pulmonary microbiota was performed using the online tool-NovoMagic at https://magic.novogene.com *via* a personal account.

The pulmonary microbiota is strongly associated with COPD. 16s rDNA gene high-throughput sequencing was employed to analyze the pulmonary microbiota to explore the mechanisms. The Chao1 index reflects the relative abundance of pulmonary microbiota, and the Shannon index reflects the diversity of pulmonary microbiota. The relative abundance of pulmonary microbiota was decreased in COPD rats. After YS treatment, the relative abundance of pulmonary microbiota showed no significant change. Meanwhile, the diversity of the pulmonary microbiota was reduced in COPD rats. (*P* > 0.05), which was significantly greater after the YS intervention (*P* < 0.05) (Figure 7A).

Next, we observed the bacteria in phylum and genera levels among the CT, COPD, and COPD + YS groups. At the phylum level, the pulmonary microbiota was dominated by Proteobacteria, Firmicutes, and Bacteroidota. It's shown that Proteobacteria and Firmicutes increased in COPD rats, while Bacteroidota decreased in COPD rats. After YS intervention, Proteobacteria and Firmicutes decreased while Bacteroidota increased (Figures 7B,C). At the genera level, the pulmonary microbiota was dominated by *Ralstonia, Mycoplasma, Halomonas, Lactobacillus, Dizetzia*, and *Bacteroides*. The COPD group showed an increased relative abundance of *Ralstonia, Mycoplasma, Halomonas*, and *Dizetzia* over the CT group. Meanwhile, there was a decreased relative abundance of *Lactobacillus* and *Bacteroides*. The relative abundance of *Halomonas, Lactobacillus, Dizetzia*, and *Bacteroides* were increased, and there was a lower relative abundance of *Ralstonia* and *Mycoplasma* after being treated with YS (Figures 7D,E).

Linear discriminant analysis of effect size showed that the iconic microorganisms in each group contributed significantly to differences in microbial structures. *Mycoplasma* was more abundant in the pulmonary microbiota of the COPD group. *Mycoplasma* was significantly increased in COPD patients. Meanwhile, *Halomonas, Dietzia*, and *Nesterenkonia* were enriched after the YS intervention. These results suggest that YS changes the relative abundance of specific bacteria and modulates the pulmonary microbiota in COPD rats (Figures 7F,G).

### Environmental Factor Correlation Analysis

**Figure 8:**
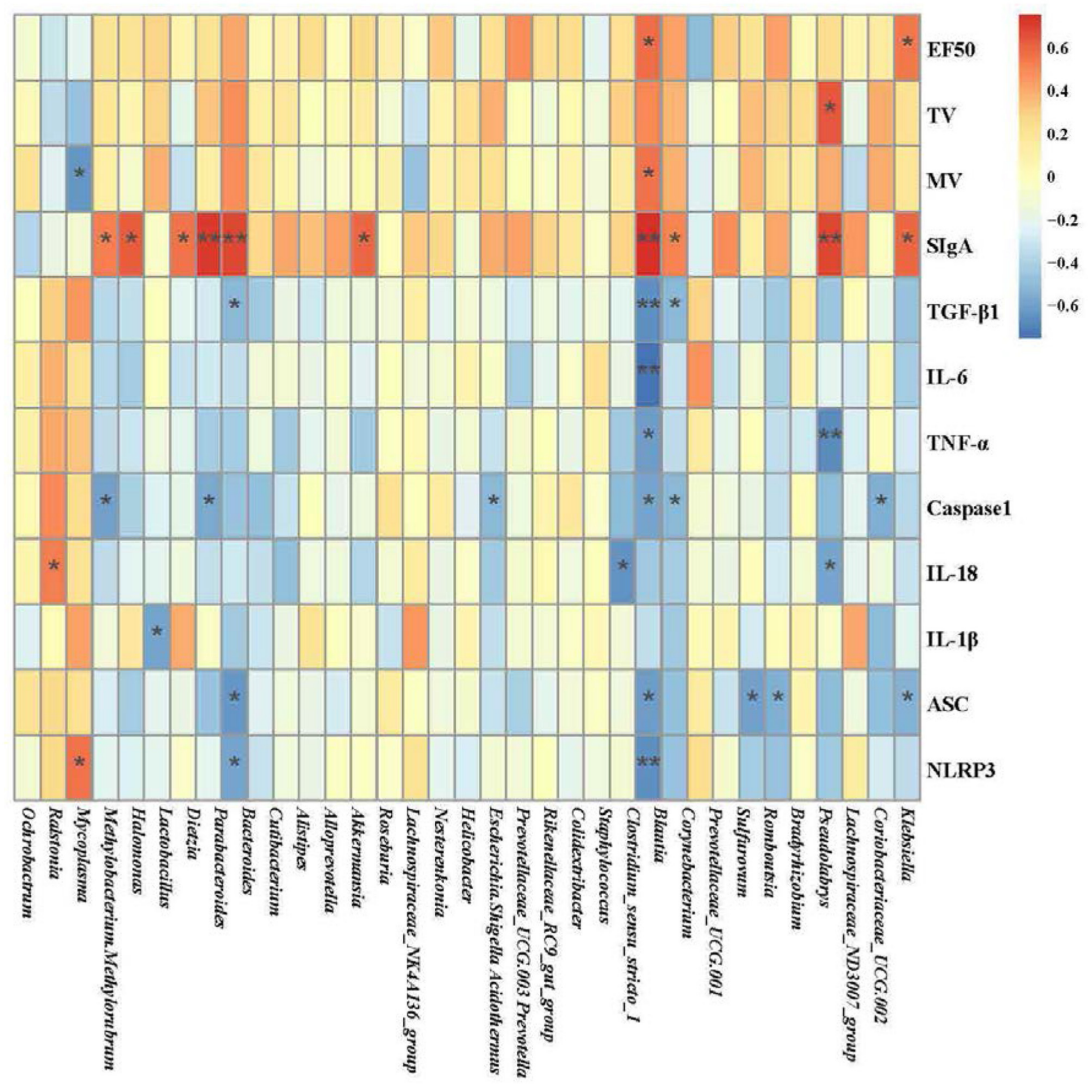
Heatmap of the correlation among the pulmonary microbiota related to environmental factors (*P < 0.05, **P < 0.01; blue represents a negative correlation; red represents a positive correlation). The analysis of pulmonary microbiota was performed using the online tool of NovoMagic at https://magic.novogene.com *via* a personal account.

Correlation heatmap plots were used to assess the top 35 genera and correlations with 12 environmental factors (EF50, TV, MV, SIgA, TGF-β1, IL-6, TNF-α, Caspase1, IL-18, IL-1β, ASC, and NLRP3). As shown in the Figure 8, EF50 was positively correlated with *Clostridium_sensu_stricto_1* and *Klebsiella* (*P* < 0.05). TV was positively correlated with *Pseudolabrys* (*P* < 0.05). MV was negatively related with *Mycoplasma* (*P* < 0.05) and positively related with *Clostridium_sensu_stricto_1* (*P* < 0.05). SIgA had a positive correlation with *Methylobacterium, Methylorubrum, Halomonas, Dietzia, Parabacteroides, Bacteroides, Roseburia, Clostridium_sensu_stricto_1, Blautia, Pseudolabrys* and *Klebsiella* (*P* < 0.05). TGF-β1 was negatively correlated with *Bacteroides, Clostridium_sensu_stricto_1* and *Blautia* (*P* < 0.05). IL-6 was negatively associated with *Clostridium_sensu_stricto_1* (*P* < 0.01). TNF-α had a negative correlation with *Clostridium_sensu_stricto_1* and *Pseudolabrys* (*P* < 0.05). Caspase1 negatively correlated with *Methylobacterium, Methylorubrum, Parabacteroides, Escherichia, Shigella, Clostridium_sensu_stricto_1, Blautia* and *Coriobacteriaceae_UCG.002* (*P* < 0.05). IL-18 had a positive correlation with *Ralstonia* (*P* < 0.05) and a negative correlation with *Staphylococcus, Pseudolabrys* (*P* < 0.05). IL-1β was negatively correlated with *Lactobacillus* (*P* < 0.05). NLRP3 was positively correlated with *Mycoplasma* (*P* < 0.05) and negatively correlated with *Bacteroides, Clostridium_sensu_stricto_1* (*P* < 0.05) (Figure 8). Among them, *Clostridium_sensu_stricto_1* was associated with more than half of the environmental factors, which may be the potential target bacteria of pulmonary microbiota for COPD progression and YS therapy. At the same time, SIgA in the lung was more correlated with pulmonary microbiota.

## Discussion

A well-known inflammation and airway remodeling cause airflow limitation, the primary pathological features of COPD (28). The infiltration of lymphocytes, neutrophils, and monocytes into the lung tissue or airway contributes to chronic inflammation (6). Clinically, neutrophilia of sputum and blood is a characteristic feature of all COPD patients (29), which is derived from chemokines including IL-1β and CXCL (30). The NLRP3 inflammasome is involved in developing diseases by promoting the release of downstream inflammatory cytokines, inducing acute and chronic inflammatory responses (31). In response to endogenous or exogenous stimuli, NLRP3 is activated. Further, ASC acts as an adaptor protein to recruit the precursor caspase-1, which has an enzymatic activity after cleaving pro-interleukin-1β and IL-18 precursor, producing mature IL-1β and IL-18, exerting proinflammatory effects (32). Superfluous inflammasome activation is a feature of numerous lung diseases, including infections (33). Excessive inflammatory factors are proven to be involved in tissue injuries and remodeling in mouse models (34). However, transgenic animal knockout IL-1β and TNF-α receptor mice are protected from developing airway remodeling (35). In the present study, the levels of NLRP3, caspase-1, ASC, IL-18, and IL-1β were decreased in YS administration animals. Insufficiently, we did not evaluate COPD sensitivity and the therapeutic effect of YS in transgenic animals of NLRP3.

Bronchodilators, anti-inflammatory agents, and hormones may improve symptoms in patients with COPD (36). The four main types (Anticholinergics, β2-Agonists, inhaled corticosteroids, and combination bronchodilator therapy) are found in GOLD reports to reduce dynamic hyperinflation at rest and during exercise and improve exercise performance (37). Antioxidant and anti-inflammatory agents were applied to treat COPD *in vitro* and *in vivo* (38). Anti-inflammatory and entire modeling effects of muscarinic receptors and antagonists were confirmed in COPD (39). All of them are important for improving symptoms. The contribution is limited in halting the progressive deterioration of pulmonary function and the progression of the disease. YS, as traditional Chinese medicine, is a clinically effective empirical formula for the treatment of lung disease, which is composed of *Hedysarum Multijugum Maxim, Atractylodes Macrocephala Koidz, Saposhnikoviae Radix, Sinapis Semen, Fritillariae Thunbrgii Bulbus, Mori Cortex, Curcumae Rhizoma*, and *Panax Notoginseng (Burk.) F. H. Chen Ex C. Chow* (19, 40). Pharmacological studies indicate that the main components of “*Hedysarum Multijugum Maxim*,” include astragalus polysaccharides (APS), flavonoids, saponins, alkaloids, and the like. Astragalus polysaccharide is the most important natural active ingredient in *Hedysarum Multijugum Maxim*, with immunomodulatory, antiviral, antifibrotic, and other pharmacological effects (41). Astragaloside IV (AS-IV) is one of the main compounds in the water extract of *Hedysarum Multijugum Maxim*. Studies have shown that AS-IV has effective protective effects on cardiovascular, lung, kidney, and brain, which may be related to its antioxidant, anti-inflammatory, and anti-apoptotic effects. It is closely related to the enhancement of immunity, the attenuation of migrations, the invasion of cancer cells, and the improvement of chemosensitivity to chemotherapeutic drugs (42). The main chemical components of *Atractylodes Macrocephala Koidz* are volatile oils, polysaccharides, lactones, and the like. Atractylodes macrocephala polysaccharide (PAMK) can improve animal immunity (43) and reduce inflammatory damage and oxidative stress in mice (44). As the main ingredient of *Saposhnikoviae Radix* (SR), SR can inhibit the release of histamine for an anti-allergic effect (45). Further, the water-soluble polysaccharides extracted and purified from SR may be one of its main anti-allergic active ingredients (46). Prim-O-glucosylcimifugin Attenuates, one of the active ingredients of *Saposhnikoviae Radix*, can attenuate endotoxin-induced inflammatory response in macrophages (47). Likewise, *Sinapis Semen* shows anti-inflammatory properties (48). *Fritillaria thunbergii Miq* and its bulbs are mainly composed of alkaloids, essential oils, diterpenoids, carbohydrates, sterols, amino acids, nucleotides, fatty acids, and lignans. Among them, Nepeta and its bulbs have a wide range of biological activities, such as anti-inflammatory, anticancer, antitussive, expectorant, antiulcer, antibacterial, antioxidant, antithyroid, regulating blood rheology, antidiarrheal, neuroprotective, and sedative pain effects (49). *Curcumae Rhizoma* has anti-platelet aggregation and antithrombotic, hepatoprotective, antioxidant, antibacterial, and antiviral activities, in which antitumor activity research is the most extensive and focused (50). Among them, curcumin, as one of the main active components of *Curcumae Rhizoma*, is a highly effective antibacterial agent effective against various types of cancer, diabetes, obesity, cardiovascular disease, lung disease, nervous system disease, and autoimmune disease (51). *Panax Notoginseng (Burk.) F. H. Chen Ex C. Chow* can reduce reactive oxygen species, lysozyme, prostaglandins, leukotrienes, and inflammatory cytokines, such as IL-1, IL-12, and TNF-α, to reduce potential damage to surrounding tissues, exerting anti-inflammatory effects (52). The main active ingredient of *Panax Notoginseng (Burk.) F. H. Chen Ex C. Chow* has been widely studied for its applications such as anti-inflammatory effects (53, 54). Pharmacologically, the function of YS has been confirmed to improve pulmonary function and ventilatory dysfunction and played an important role in repairing inflammatory damage in COPD animals.

The study of the airway microbiota is in its infancy. Still, it has been gradually recognized that the microbiota of the respiratory tract is associated with pulmonary disease (55). Abundant microbial diversity is regarded as beneficial in development and immune system domestication, affecting the susceptibility to allergic asthma, cystic fibrosis, respiratory infection, and lung cancer (56–58). Current reports suggest that the development of COPD is primarily achieved through microbiota. However, risk factors for COPD, including cigarette smoke and inhaling contaminated air, can affect the composition of the pulmonary microbiota (59). In contrast to healthy individuals, the influence of the lung microenvironment on the microbiome in COPD could be obvious (60). The diversity of the sputum microbiome is decreased in COPD patients (61), such as *Haemophilis influenzae* increasing at the exacerbation of COPD (62). We observed lower microbial alpha diversity in the COPD group than in the control group. The lung microbial alpha diversity of mice in the COPD+YS groups differed from that of the COPD group. Analysis of flora diversity showed that YS prevented pulmonary microbiota dysbiosis in COPD rats. Consistent with this finding, linear discriminant analysis of effect size showed different lung microbial compositions in these groups. *Mycoplasma* belongs to *Firmicutes*, which accelerates disease processes by promoting chronic inflammation (63). *Halomonas* are gram-positive bacteria. Various oligotypes of *Christensenella* and *Clostridium* were detected in lung tissue and a few oligotypes of *Halomonas* in both airway fluid and lung tissue from pulmonary fibrosis and lung cancer patients, suggesting the involvement of these microbial communities in fibrotic diseases (64). *Nesterenkonia* belongs to *Actinobacteria* and *Lactobacillaceae*. The microbiome might play a significant role in maintaining normal lung function, including structural and immune barriers (65). These findings suggest that in COPD treatment, the relative abundance of *Halomonas* and *Nesterenkonia* are increased by decreasing the relative abundance of *Mycoplasma*, potentially inhibiting the inflammatory factors IL-1β, TNF-α, IL-6, IL-1β, and TGF-β1. This further increases SIgA expression, which enhances immunity, alleviates collagen deposition and improves lung function. We used the correlation heatmap plots to assess the top 35 genera and correlations with 12 environmental factors (EF50, TV, MV, SIgA, TGF-β1, IL-6, TNF-α, Caspase1, IL-18, IL-1β, ASC, and NLRP3) to further explore the relationship between pulmonary microbiota and pulmonary ventilation function, inflammatory factors, and immunity. Clostridium_sensu_stricto_1 may be the potential target bacteria of pulmonary microbiota for COPD progression and YS therapy. Besides, SIgA in the lung may be an important factor affecting the pulmonary microbiota.

## Summary and Prospects

In this work, we confirmed the function of YS in improving the pulmonary ventilatory function in COPD rats referring to EF50, TV, and MV. Histopathological findings showed that inflammatory injury associated with COPD was weakened. The inflammatory mediators of NLRP3/caspase-1/IL-1βsignaling were involved in pulmonary pathological damage. Furthermore, disturbed pulmonary microbiota was discovered in COPD animals. However, after administration with YS, the abundance and diversity of pulmonary microflora were remodeled, especially increasing the beneficial genua. The replacement of flora is associated with inflammation by environmental factor correlation analysis. In general, we provided some evidence that dysbiosis of pulmonary microbiota leads to the COPD pathological process. The function of the Yifei Sanjie Formula was involved in remodeling lung microbes and anti-inflammatory signal pathways. To evaluate the sensitivity of COPD or pharmacodynamic tests is positive in animals with germ-free lungs, which is verified for further study in the future. It is certain targeting intervention microbiota to anti-inflammatory may be a potential therapeutic strategy for COPD.

## Data Availability Statement

The datasets presented in this study can be found in online repositories. The names of the repository/repositories and accession number(s) can be found in the article/Supplementary Material.

## Ethics Statement

The animal study was reviewed and approved by the Animal Ethics Experiment Committee of Yunnan University of Traditional Chinese Medicine. Written informed consent was obtained from the owners for the participation of their animals in this study.

## Author Contributions

ZY and JY: study design. YW: data collection. YW, HM, and BQ: statistical analysis and data interpretation. NL, QZ, WJ, HX, and YL: manuscript preparation. ZY, JY, and YW: funds collection. All authors contributed to the article and approved the submitted version.

## Funding

This work was funded in part by a grant from the National Natural Science Foundation of China (81860646, 81760819), Yunnan Provincial Science and Technology Department (202201AS070084, 202005AC160058, 202101AZ070001-012, 202005AF150063, 202102AE090031), the Science Research Foundation of Yunnan Provincial Department of Education (2020Y0204), Yunnan Science and Technology Planning Project-Joint Major Project of Traditional Chinese Medicine (2019FF002[-002]), and the Provincial Innovation Team of the Yunnan University of Chinese Medicine for Traditional Chinese Medicine to Regulate Human Microecology (No. 2018HC011).

## Conflict of Interest

The authors declare that the research was conducted in the absence of any commercial or financial relationships that could be construed as a potential conflict of interest.

## Publisher's Note

All claims expressed in this article are solely those of the authors and do not necessarily represent those of their affiliated organizations, or those of the publisher, the editors and the reviewers. Any product that may be evaluated in this article, or claim that may be made by its manufacturer, is not guaranteed or endorsed by the publisher.
